# Brain enhancer activities at the gene-poor 5p14.1 autism-associated locus

**DOI:** 10.1038/srep31227

**Published:** 2016-08-09

**Authors:** Yukiko U. Inoue, Takayoshi Inoue

**Affiliations:** 1 Department of Biochemistry and Cellular Biology, National Institute of Neuroscience, National Center of Neurology and Psychiatry, Ogawahigashi, 4-1-1, Kodaira, Tokyo 187-8502, Japan

## Abstract

Due to the vast clinical and genetic heterogeneity, identification of causal genetic determinants for autism spectrum disorder (ASD) has proven to be complex. Whereas several dozen ‘rare’ genetic variants for ASD susceptibility have been identified, studies are still underpowered to analyse ‘common’ variants for their subtle effects. A recent application of genome-wide association studies (GWAS) to ASD indicated significant associations with the single nucleotide polymorphisms (SNPs) on chromosome 5p14.1, located in a non-coding region between cadherin10 (*CDH10*) and cadherin9 (*CDH9*). Here we apply an *in vivo* bacterial artificial chromosome (BAC) based enhancer-trapping strategy in mice to scan the gene desert for spatiotemporal *cis*-regulatory activities. Our results show that the ASD-associated interval harbors the cortical area, striatum, and cerebellum specific enhancers for a long non-coding RNA, moesin pseudogene1 antisense (*MSNP1AS*) during the brain developing stages. Mouse moesin protein levels are not affected by exogenously expressed human antisense RNAs in our transgenic brains, demonstrating the difficulty in modeling rather smaller effects of common variants. Our first *in vivo* evidence for the spatiotemporal transcription of *MSNP1AS* however provides a further support to connect this intergenic variant with the ASD susceptibility.

Autism spectrum disorder (ASD) represents an extremely heterogeneous neurodevelopmental syndrome with a complex genetic etiology. While dozens of rare mutations and copy number variations with large effects on ASD susceptibility have been identified in the past decade, studies have been underpowered to analyse common polymorphisms with rather smaller effects^1^,^2^,^3^. A genome-wide association study (GWAS), designed to identify common genetic risk factors underlying ASD, has revealed strong association signals on chromosome 5p14.1 located in a 2.2 million base pairs (bp) gene desert between cadherin10 (*CDH10*) and cadherin9 (*CDH9*)^4^. The most statistically significant association peaked at rs4307059 (*P* = 10^−10^), which was flanked by five other SNPs that all achieved genome-wide significance (P < 10^−8^), indicating that the association at rs4307059 was not due to a technical artifact. The six risk SNPs reside within the same ~100 kb linkage disequilibrium (LD) block, implicating that theses SNPs are tagging the same variants. The common genetic variant, rs4307059, was also associated with social communication phenotypes in general population samples, suggesting that this genetic signal might be a quantitative trait locus for ASD-related social communication phenotypes^5^.

Although the nearest protein-coding genes, *CDH10* and *CDH9*, encode cell-cell/synapse adhesion molecules that have crucial roles in neural development^6^, the lack of correlation between rs4307059 genotypes and expression levels of these genes in postmortem adult brain has been described^4^,^7^. On the other hand, it has been reported that a long non-coding RNA antisense to moesin (*MSN*) is transcribed directly at the chromosome 5p.14.1 ASD-associated GWAS site^7^. When over-expressed in cultured human cell lines, the 3.9-kb non-coding RNA, *MSNP1AS* (moesin pseudogene 1 antisense) was demonstrated to bind to the transcript of the X chromosome protein-coding gene *MSN* and negatively regulate MSN protein expression^7^. In addition, the ASD-associated rs4307059 T allele showed increased expression of the antisense RNA in postmortem temporal cortex of individuals with ASD^7^. However, these data could not fully explain the involvement of this antisense RNA in the ASD risk. Because both expressions of *CDH10*/*CDH9* and *MSNP1AS* were all quantified by using postmortem adult brain tissues, we don’t know yet how the expression levels correlated with the ASD susceptibility during embryonic and/or early postnatal brain development. The presence of *MSNP1AS* expressions in the developing brains has not been confirmed yet, either.

In this study, we hypothesise that the 5p14.1 ASD GWAS peak would indicate the presence of transcriptional regulatory activity in the brain developing stages. To this end, we apply the bacterial artificial chromosome (BAC) based enhancer-trapping strategy in mice^8^ to efficiently screen the *cis*-regulatory activities in the target gene desert. We accordingly demonstrate that the 5p14.1 ASD-associated interval actually harbors the cortical area, striatum, and cerebellum specific enhancers, and our BAC transgenic mouse line does express the antisense RNA, *MSNP1AS*, in crucial developmental periods for ASD etiology.

## Results

### Bacterial artificial chromosome (BAC) selection and modification for enhancer-trapping scan at the 5p14.1 ASD-associated locus

**Figure 1:**
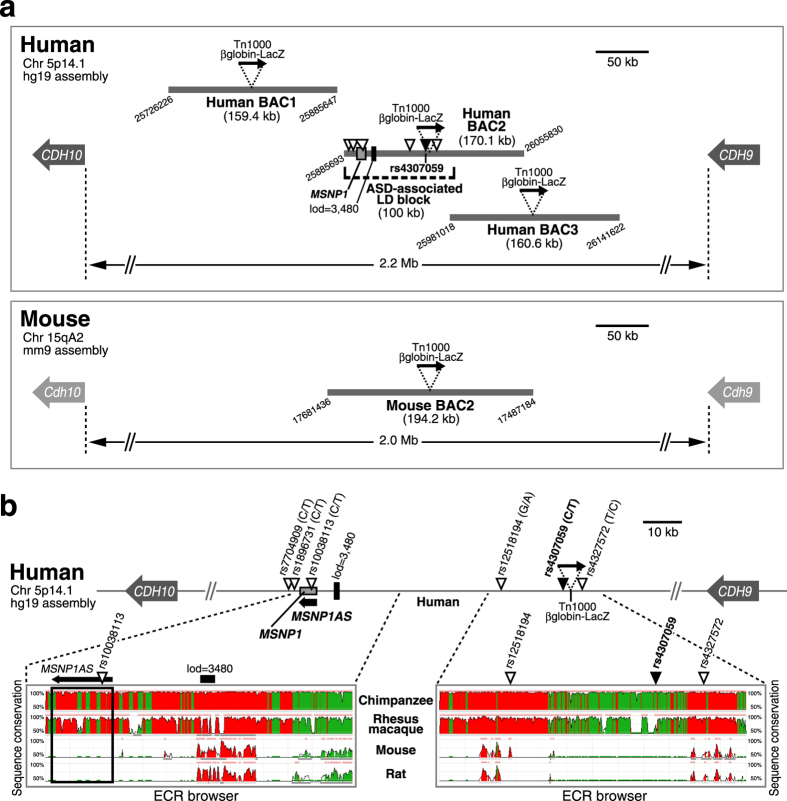
Schematic overview of the intergenic BACs used in enhancer-trapping for the 5p14.1 ASD-associated gene desert. (**a**) The upper panel shows human intergenic region between *CDH10* and *CDH9*, apart from 2.2 megabase (Mb) on the chromosome 5p14.1. Three Human BACs depicted by bold gray lines are chosen for this study. The downward triangles on Human BAC2 indicate ASD-associated GWAS SNPs and particularly the black one denotes the most significant association SNP, rs4307059. All the risk SNPs reside within the same 100 kilobase (kb) linkage disequilibrium (LD) block. Only Human BAC2 covers all the six risk SNPs and moesin pseudogene 1 (*MSNP1*), whereas the adjacent Human BAC1 and Human BAC3 do not contain them. The attached digits at the edges of BACs mean the chromosomal location by UCSC Genome Browser hg19 assembly. All the BACs are tagged by *in vitro* transposition of Tn1000 βglobin-LacZ (black arrow) for enhancer-trapping. Lod score 3,480 on Human BAC2 represents a highly conserved genomic element by phastCons in UCSC Genome browser. The lower panel shows mouse intergenic region between *Cdh10* and *Cdh9*. Mouse BAC2 equivalent to Human BAC2 is selected in consideration of the evolutional conservation to the maximum extent, and also tagged by βglobin-LacZ cassette. The attached digits represent the chromosomal location by UCSC Genome Browser mm9 assembly. (**b**) In the upper column, (minor/major allele) is shown for each SNP. As an example, for rs4307059, the minor allele is C and the major allele is T. Tn1000 βglobin-LacZ gene cassette never override any risk SNPs. A 3.9 kb non-coding RNA, moesin pseudogene 1 antisense (*MSNP1AS,* bold black arrow) is encoded by the opposite (antisense) strand of *MSNP1*. In the lower column, bioinformatic analyses reveal that the human ASD risk SNPs’ regions are highly conserved among primates but not in rodents, particularly for the sequence from which *MSNP1AS* is transcribed (the region enclosed by the black rectangular line). Images were modified from ECR browser (ecrbrowser.decode.org). The vertical axes visualize the significant conservation from 50% to 100% identity. Red colored peaks are within intergenic regions, and peaks colored in green correspond to transposable elements or simple repeats.

The 5p14.1 ASD-associated GWAS peak had been mapped ~1 million bp from either the nearest cadherin gene. Since the LD block encompassing the risk SNPs contained a highly conserved genomic element (log odds score = 3,480 by phastCons in UCSC Genome browser in Fig. 1a), it was hypothesised that these SNPs were capturing the association of functional variants that regulate the expression of either *CDH10* or *CDH9* from a long distance^4^. Contrary to their expectation, there was no correlation between the ASD-associated marker genotypes and expression of either *CDH10* or *CDH9* at least in the adult postmortem brains^4^,^7^, retaining the possibility that the SNP genotypes affect the expression levels of those genes only at the embryonic and/or developmental stages.

To examine the ASD-associated non-coding region for regulatory activities in the context other than the limited human resources, we used BAC scan approach in an *in vivo* mouse transgenic reporter assay^8^. This strategy could allow the rapid and efficient scanning of large genomic intervals for *cis*-regulatory elements, and be readily applied to any locus of interest. We accordingly utilized three human BACs shown in Fig. 1a, which together span 415.4 kb. Human BAC2 (RP11-261G10) covered the entire ASD-associated LD block (100 kb), including the six risk SNPs and moesin pseudogene 1 (*MSNP1*) depicted in Fig. 1b. By sequencing, all the SNPs in Human BAC2 were confirmed to be the major version (risk version). For instance, SNP rs4307059 (C/T), the variant exhibiting the strongest association to ASD was common T version. On the other hand, Human BAC1 (RP11-140M9) and Human BAC3 (RP11-99D18) were selected not to contain the ASD-associated LD block (Fig. 1a).

We next employed a web-based tool “ECR browser” (ecrbrowser.decode.org) to compare multiple mammalian genome sequences corresponding to Human ASD-associated locus and examined the evolutional conservation. As a result, the associated region was highly conserved among primates (Chimpanzee and Rhesus macaque) but not in rodents (Mouse and Rat), particularly for the sequences from which *MSNP1AS* was transcribed (Fig. 1b). Hence we selected Mouse BAC2 equivalent to Human BAC2 in consideration of its evolutional conservation to the maximum extent for analysing the enhancer activities (Fig. 1a).

Each BAC was tagged through a Tn1000 transposon-mediated random insertion of a β-galactosidase (*LacZ*) gene driven by a β-globin minimal promoter^9^, and the insertion sites were determined by sequencing^9^. In Human BAC2 clone, Tn1000 βglobin-LacZ cassette was inserted at chr5: 25968832, which is between rs4307059 (chr5: 25967453) and rs4327572 (chr5: 25972571), not overriding any risk SNPs (Fig. 1b). More simply, the reporter cassette was integrated at 1,379 bp upstream from rs4307059 and 3,739 bp downstream from rs4327572. Through this type of BAC modification, the reporter cassette functions as an enhancer trap capturing any long-range enhancers contained in the BAC that can act upon the β-globin minimal promoter to drive region- and temporal-specific *LacZ* expression^8^,^9^. Each reporter modified BAC was then injected into fertilized mouse eggs to generate stable transgenic mouse (Tg) lines.

### Only Human BAC2 encompassing the ASD-associated SNPs captured brain enhancer activities

**Figure 2:**
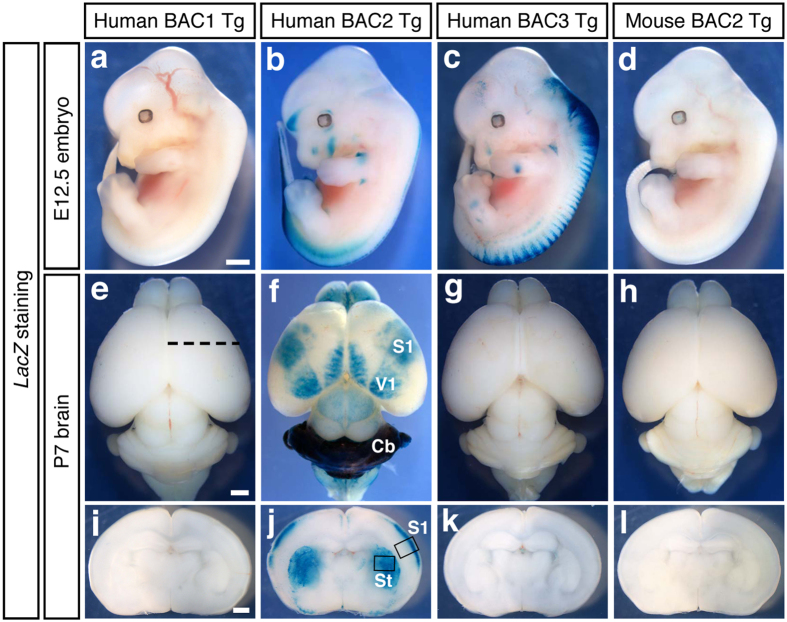
Only Human BAC2 Tg mice carrying the ASD GWAS SNPs trap brain enhancer activities. (**a**–**d**) Lateral views of the whole-mount *LacZ* stained embryos at E12.5. Human BAC2 Tg (**b**) and Human BAC3 Tg (**c**) capture enhancer activities mainly in dorsal side of the embryonic spinal cord. Scale bar, 1 mm. (**e**–**h**) Dorsal views of the whole-mount *LacZ* stained brains at P7. The rostral sides of the brains are arranged at the upper part of the panels. Only Human BAC2 Tg (**f**) shows *LacZ* reporter expression specific to the developing cerebral cortex and cerebellum. S1; primary somatosensory cortex, V1; primary visual cortex, Cb; cerebellum. Mouse BAC2 (**h**) equivalent to Human BAC2 (**f**) does not harbor any brain enhancer activities. Scale bar, 1 mm. (**i**–**l**) Coronal sections at the black dotted line levels in (**e**) at P7. Only Human BAC2 Tg (**j**) captures *LacZ* reporter expression in the striatum (St) and particular layers of the somatosensory cortex (S1). The brain tissues enclosed by the black lines are used for immunohistochemistory in Fig. 3. Mouse BAC2 (**l**) corresponding to Human BAC2 (**j**) captures enhancer activities neither in striatum nor in cortical layers. Scale bar, 1 mm.

First, we analysed the *LacZ* expressions of Human BAC Tg lines at embryonic day 12.5 (E12.5). Human BAC2 Tg (Fig. 2b) and Human BAC3 Tg (Fig. 2c) captured enhancer activities mainly in dorsal sides of the embryonic spinal cord, but Human BAC1 Tg (Fig. 2a) did not show any *LacZ* signals. Then we scanned the enhancer activities of the Human BAC Tg lines at postnatal day 7 (P7) in the whole brains and in the coronal sections. As the results, only Human BAC2 Tg (Fig. 2f,j) captured *LacZ* reporter expressions specific for the cerebral cortex, cerebellum (Cb), and striatum (St). In the cerebral cortex, *LacZ* signals clearly corresponded to primary somatosensory cortex (S1) and primary visual cortex (V1). On the other hand, Human BAC1 Tg (Fig. 2e,i) and Human BAC3 Tg (Fig. 2g,k) did not harbor any brain enhancer activities. As the region-specific *LacZ* expressions were observed only in Human BAC2 but not in the overlapping Human BAC3, the enhancers should reside within the non-overlapped 95.3 kb interval (Fig. 1a), meaning that the 100 kb LD block spanning all the risk variants might drive *cis*-regulatory activities in the particular areas of cerebral cortex, striatum, and cerebellum. Although the existence of more diffuse, subtle problems in the brain structure and function across the entire brains in ASD individuals have been accumulated in the literature, these structures are repeatedly reported to have correlative changes with the disease^10^,^11^,^12^,^13^,^14^,^15^.

Since all the six SNPs associated with ASD risk in Human BAC2 were confirmed to be the risk versions by direct sequencing, the brain enhancer activities we captured in this scan were for the risk genotypes.

In addition, we found that Mouse BAC2 equivalent to Human BAC2 (Fig. 1a) did not harbor any enhancer activities (Fig. 2d,h,l). Because the ASD-associated interval was highly conserved among primates but not in rodents (Fig. 1b), it is suggested that the *cis*-regulatory modules captured in Human BAC2 could be unique for primates.

### Enhancer activities at 5p14.1 ASD-associated locus were localised in the S1 cortical layer II/III, striatal, and cerebellar neurons

**Figure 3:**
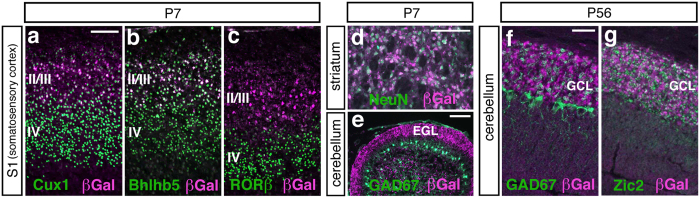
In Human BAC2 Tg, the enhancer activities are localised in the S1 cortical layer II/III, striatal, and cerebellar neurons. (**a**–**c**) The tissue sections from somatosensory cortex (S1) enclosed by the black line in Fig. 2j are immuno-stained. In the somatosensory cortex (S1) at P7, the βGal expressing neurons also express Cux1 (**a**) and Bhlhb5 (**b**) both known as upper layer markers, but they do not co-stained with the layer IV marker, RORβ (**c**), meaning that the enhancer activities delineate the cortical layer II/III. Scale bar, 100 μm. (**d**) The striatum tissues (St) enclosed by the black line in Fig. 2j are immuno-stained at P7. Almost all the βGal expressing cells are co-stained with the differentiated neuron marker, NeuN. Scale bar, 100 μm. (**e**) The cerebellum tissues (Cb) in Fig. 2f are immuno-stained at P7. The βGal expressing cells reside mainly in the external granular layer (EGL) above the Purkinje cell layer marked with GAD67 expression. Scale bar, 100 μm. (**f**,**g**) In the cerebellum at P56, the βGal expressing cells are present in the granule cell layer (GCL) and are co-stained with Zic2, a granule cell marker. Scale bar, 50 μm.

To precisely probe the cell types that harbored enhancer activities in Human BAC2 Tg, we prepared the brain tissue samples and stained with antibodies for β-galactosidase (βGal) and marker molecules. First, the tissues from somatosensoty cortex (S1) enclosed by the black line in Fig. 2j were immuno-stained to determine which layer contains the βGal expressing cells. At P7, the βGal expressing cells co-expressed Cux1 (Fig. 3a) and Bhlhb5 (Fig. 3b) both known as upper layer markers, but they were not co-stained with the layer IV marker, RORβ (Fig. 3c), demonstrating that the enhancer activities resided in layer II/III cortical neurons. Then, the striatum (St) tissues enclosed by the black line in Fig. 2j were immuno-stained at P7. Almost all the βGal expressing cells co-stained with the differentiated neuron marker, NeuN (Fig. 3d). Next, the cerebellum (Cb) tissues in Fig. 2f were stained at P7. The βGal expressing cells existed mainly in the external granular layer (EGL) above the Purkinje cell layer marked with GAD67 expression (Fig. 3e). In the cerebellum at P56, the βGal expressing cells were present in the granule cell layer (GCL) (Fig. 3f) and co-localised with Zic2, a granule cell marker (Fig. 3g). Collectively, the enhancer activities at the 5p14.1 ASD-associated interval tended to be localized at neurons in those brain structures reported to have the implication with the disease^10^,^11^,^12^,^13^,^14^,^15^. Incidentally, callosal projection neurons in upper cortical layers (II/III), whose axons make up the corpus callosum connecting the two cerebral hemispheres, play key roles in high-level associative connectivity and their dysgenesis is reported to be one of only a few reproducibly identified pathologies in ASD^16^. The upper layer-specific enhancer activities we detected are thus remarkable on this point.

### *MSNP1AS* was confirmed to be transcribed in Human BAC2 Tg mouse brains and the expression level well correlated with that of *LacZ* mRNA

**Figure 4:**
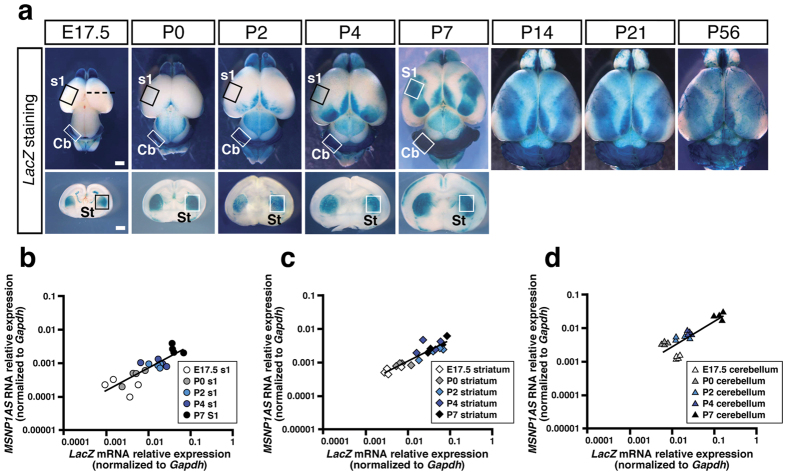
*MSNP1AS* is actually transcribed in Human BAC2 Tg mouse brains and the expression levels are well correlated with those of *LacZ* mRNA. (**a**) Developmental *LacZ* expression patterns in Human BAC2 Tg brains. *LacZ* expressions in the cerebral cortex gradually become thicker and broader from E17.5 to P7, and are maintained to the adult stage (P14, P21, P56). At E17.5, the black dotted line in the upper panel corresponds to the coronal section level for the lower panel. *LacZ* expressions in the cerebellum (Cb) and striatum (St) are also kept at considerable levels through all the stages analysed. Total RNA samples are extracted from boxed areas tagged with S1 (s1), St and Cb from E17.5 to P7. S1; primary somatosensory cortex, s1; prospective regions for S1, St; striatum, Cb; cerebellum. Scale bars, 1 mm for whole brain and coronal section. (**b**) *MSNP1AS* transcript levels are positively associated with *LacZ* transcript levels in S1 (s1) samples from E17.5 to P7 (*r* = +0.75; *P* = 1.20 × 10^−4^). Data are shown relative to *Gapdh* expression. (**c**) Association of *MSNP1AS* transcript levels with *LacZ* transcript levels in the striatum (St) from E17.5 to P7 (*r* = +0.81; *P* = 1.33 × 10^−5^). Data are shown relative to *Gapdh* expression. (**d**) Association of *MSNP1AS* transcript levels with *LacZ* transcript levels in the cerebellum (Cb) from E17.5 to P7 (*r* = +0.93; *P* = 1.15 × 10^−9^). Data are shown relative to *Gapdh* expression.

To know the developmental change of *LacZ* expression patterns in Human BAC2 Tg, we stained whole brain samples collected from E17.5 to P56 (Fig. 4a, upper column) and coronal sections from E17.5 to P7 (Fig. 4a, lower column). As the results, we found that *LacZ* expressions in the cerebral cortex gradually became thicker and broader from E17.5 to P7 and were maintained to the adult stage (P14, P21, P56). *LacZ* expressions in the cerebellum and striatum were also kept at considerable levels through all the stages analysed. These results indicated that the brain enhancers at 5p14.1 ASD-associated interval were continuously in action from the embryonic to the adult stage.

To determine whether primate specific *MSNP1AS* is actually transcribed from the ASD GWAS signal site in our Human BAC2 Tg mice, we next performed quantitative PCR assays on total RNA prepared from the brain samples. We extracted total RNAs from the boxed regions tagged with the somatosensory cortex (S1 or s1), striatum (St) and cerebellum (Cb) from E17.5 to P7 (Fig. 4a). We then used TaqMan probes for *MSNP1AS* and *LacZ* to compare those expressions with each other. The quantitative assays revealed that *MSNP1AS* was indeed expressed at detectable ranges (expression relative to *Gapdh* ranged from 0.0001 to 0.03) although its expression level was lower than the *LacZ* mRNA expression level (Fig. 4b–d). Not surprisingly, *MSNP1AS* and *LacZ* mRNA were not detected (Ct >40 cycles) in wild type, Human BAC1 Tg, Human BAC3 Tg, and Mouse BAC2 Tg mouse brain tissues. By calculating Pearson correlation coefficient (*r*) and *P* value between *LacZ* mRNA expression and *MSNP1AS* RNA expression in Human BAC2 Tg mouse brain tissues, we found the significant correlation between them (Fig. 4b–d). These data indicated that the brain enhancer activities detected in Human BAC2 Tg mice might regulate *MSNP1AS* expression.

In addition to the *LacZ* blue-stained region (S1; somatosensory cortex), we sampled two regions with little *LacZ* staining at P7 (A; auditory cortex, H; hypothalamus in Supplementary Fig. S1-a) as the reference and quantified the *LacZ* mRNA/*MSNP1AS* expression levels to confirm the ‘region-specific’ enhancer activities in Human BAC2 Tg mouse brains. As the results, the *LacZ* mRNA/*MSNP1AS* transcript levels in blue-coloured S1 were higher than those in the non-stained A and H (Fig. S1-b, S1-c), indicating that the transcription was enhanced in blue-stained regions.

When we carefully observed the panel Fig. 4a at P7, the concentration of blue colour in the cerebellum was thicker than that in the somatosensory cortex/striatum. Correspondingly the *LacZ* mRNA/*MSNP1AS* expression levels in cerebellum were one-order higher than those in somatosensory cortex/striatum, well quantifying the feature of region specific enhancer activities. Similarly, temporal specificity of the S1 (s1) enhancer well correlated with our quantitative data (Fig. 4a,b).

### *Msn* mRNA and protein levels were almost unchanged in Human BAC2 Tg mouse brains

**Figure 5:**
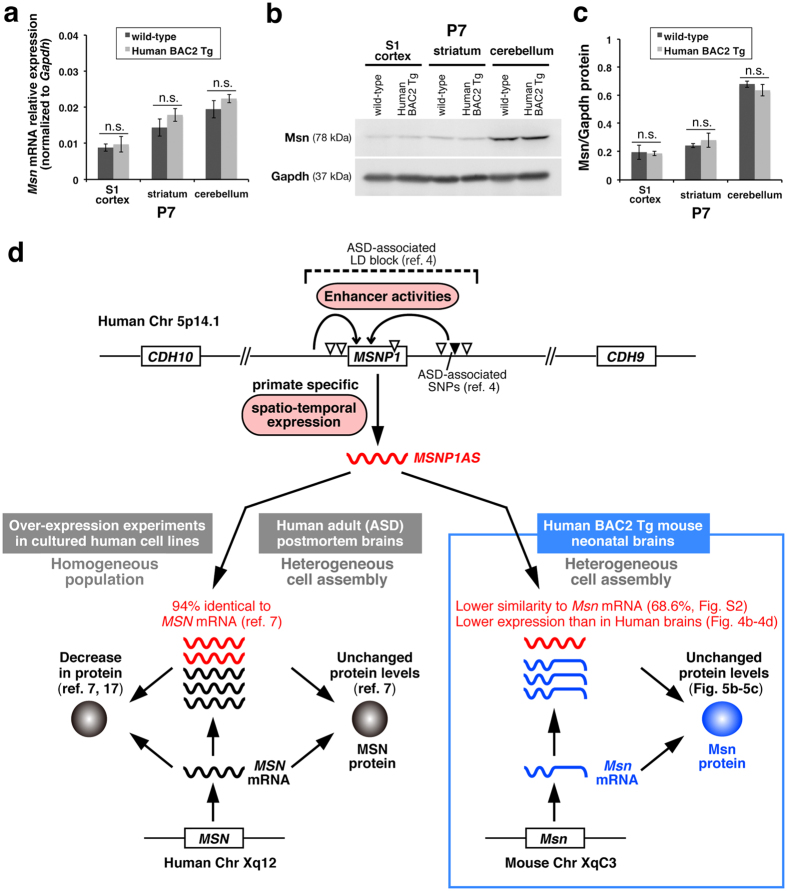
*Msn* mRNA and protein levels are almost unchanged in Human BAC2 Tg mouse brains. (**a**) Exogenously expressed *MSNP1AS* does not largely affect on the *Msn* mRNA amounts in Hunam BAC2 Tg mouse brains. *Msn* transcript levels in the primary somatosensory cortex (S1 cortex), striatum, and cerebellum from Human BAC2 Tg at P7 are slightly increased but almost similar to those in wild-type mice. Data are shown relative to *Gapdh* expression. n.s.; not significant. (**b**) Representative Western blots for Msn and Gapdh to the protein samples from the S1 cortex, striatum, and cerebellum in wild-type and Human BAC2 Tg mice at P7. (The original whole image can be found as Supplementary Fig. S3). (**c**) Quantitative summary of Msn protein expressions in (**b**). Exogenous *MSNP1AS* does not reduce the Msn protein levels in Human BAC2 Tg mouse brains. n.s.; not significant. (**d**) Schematics of brain enhancer activities for *MSNP1AS* at 5p14.1 ASD-associated region. The enhancer activities we detected in Human BAC2 Tg mouse brains are depicted as the curved arrows in the upper column. The downward triangles indicate ASD-associated SNPs^4^. Compared to the 94% identity between *MSNP1AS* and *MSN* mRNA in human cells^7^, mouse *Msn* mRNA has lower similarity to *MSNP1AS* (68.6% matches at the maximum, see Supplementary Fig. S2). Although over-expression of *MSNP1AS* in almost homogeneous population of human cell lines decreases MSN protein levels^7^,^17^, total MSN protein is unchanged in the heterogeneous postmortem brain (=mixture of *MSN* mRNA/*MSNP1AS* positive and negative cell assembly) from individuals with ASD^7^. In Human BAC2 Tg mice, Msn protein levels also remain unaffected just like the case with the human postmortem brains. In addition, expression levels of the exogenously expressed *MSNP1AS* in Human BAC2 Tg mouse brains (Fig. 4b–d) tend to be lower than those of endogenous *Msn* mRNA (Fig. 5a), whereas the antisense RNA levels are relatively similar to *MSN* mRNA levels in the human postmortem brains^7^. This might further reason the unchanged Msn protein levels in Human BAC2 Tg.

Since it was reported that *MSNP1AS* (NT_006576.16) had 94% sequence identity in the reverse complement to Human *MSN* mRNA (NM_002444.2) and its over-expression in cultured human cells caused decreases in MSN protein levels^7^,^17^ (Fig. 5d), we aligned *MSNP1AS* sequence with the reverse complement of mouse *Msn* mRNA (NM_010833.2). As a result, we found that *MSNP1AS* had 68.6% matching possibility to mouse *Msn* mRNA complement (see Supplementary Fig. S2). We therefore hypothesised that *MSNP1AS* might not affect on endogenous *Msn* mRNA and protein levels in Human BAC2 Tg mouse brains. To rigorously compare the *Msn* mRNA amounts between wild-type and Human BAC2 Tg mouse brains, we performed quantitative PCR assays on the total RNA samples (P7) extracted from the boxed areas in Fig. 4a. Consequently, the *Msn* mRNA amounts in the somatosensory cortex (S1), striatum, and cerebellum in Human BAC2 Tg at P7 were slightly increased but almost similar to those in wild-type mice (Fig. 5a). In addition, to know whether exogenous *MSNP1AS* in Human BAC2 Tg mouse brain affect on the Msn protein amounts, we performed Western Blot analyses with anti-Msn antibodies to protein samples extracted from the somatosensory cortex (S1), striatum, and cerebellum at P7 (Fig. 5b, the original whole image can be found as Supplementary Fig. S3). As the results, we detected no significant differences in Msn protein between Human BAC2 Tg and the control wild-type mice (Fig. 5c). Hence, we confirmed that exogenously expressed *MSNP1AS* in Human BAC2 Tg mouse brains has little influence on the Msn protein amount (Fig. 5d).

Incidentally, we didn’t observe distinct morphological changes in the Tg mouse brains at P7 (see Supplementary Fig. S4). Additionally, we didn’t detect abnormal behaviours suggestive of neurodevelopmental disorders: among 149 adult Tg mice we observed, there was no evidence for systemic tremors or epileptic seizures. These adult Tg mice had no problems in sexual communication and never showed repetitive self-grooming. Among 370 Tg pups (P0–P7) we obtained, the deliveries were always normal without any premature birth. For these pups, neglected nursing did not occur, implying their ultrasonic vocalization might be normal. Besides, among 67 Tg embryos studied, we never found early prenatal abnormalities.

As was already shown in Fig. 3, we confirmed typical staining patterns of various neuronal cell markers in the somatosensory cortex (i.e. Cux1, Bhlhb5, RORβ), striatum (NeuN) and cerebellum (Zic2), indicating that our Human BAC2 Tg mice at least develop normal cytoarchitecture in the brain. Moreover the usual staining pattern of a neuronal activity dependent transcription factor RORβ in the Tg somatosensory cortex would suggest synaptic connections at this territory (i.e. layer IV) are virtually normal in the Tg mice.

### A forebrain enhancer for *CDH10*, one of the nearest genes from the ASD associated SNPs, mainly resided in the vicinity of the gene

*CDH10* and *CDH9,* the nearest protein-coding genes from the ASD GWAS peak, encode type II classic cadherins that mediate cell-cell/synapse adhesion, playing crucial roles in various steps of neural development^6^. In the original GWAS report, Wang *et al.* assessed the expression of *CDH10* and *CDH9* in the normal human fetal brain at the mid-gestation and found that while *CDH9* was expressed uniformly at low levels and uninformative in the nervous system, the *CDH10* expression was robust in the frontal cortex^4^. Expression patterns for *CDH10* were similar to those for *CNTNAP2*^18^ and *AUTS2*^19^, molecules now well-established to be involved in the ASDs. Moreover, neuroanatomical studies had repeatedly implicated abnormal brain development on the frontal lobes in ASD. In consideration of these findings, we lastly focused on the frontal *CDH10* expression pattern in developing brains.

**Figure 6:**
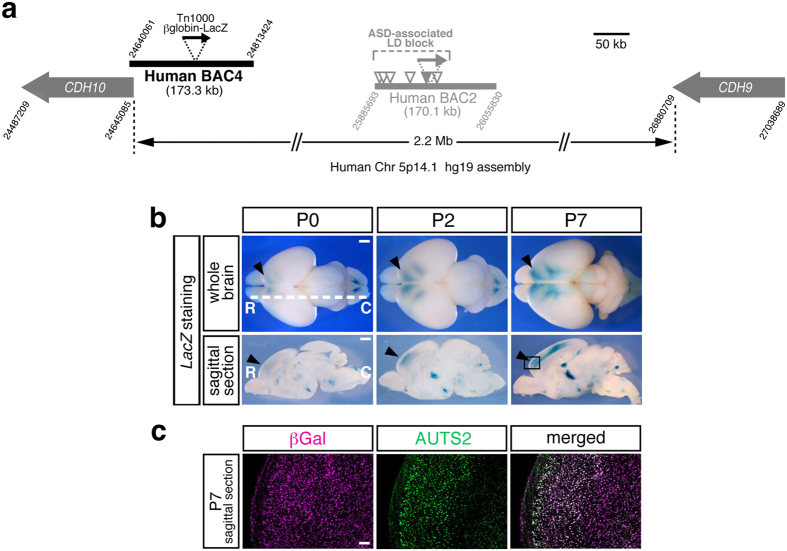
The additional Human BAC4 Tg reveals that a forebrain enhancer for *CDH10*, one of the nearest genes from the ASD risk SNPs, resides in the vicinity of the gene. (**a**) Schematic overview of Human BAC4 covering the 5′ upstream region of *CDH10* gene. The attached digits at the edges of BACs and genes mean the chromosomal location by UCSC Genome Browser hg19 assembly. Human BAC4 is tagged by Tn1000 βglobin-LacZ (black arrow) for enhancer-trapping and is processed to transgenic mice generation. (**b**) Dorsal views of the *LacZ* stained whole brains are arranged in the upper column and the sagittal sections at the white dotted line levels are arranged in the lower column. R; rostral, C; caudal. Human BAC4 Tg captures the human specific *CDH10* expression pattern enriched in the forebrain at the early developmental stages (from P0 to P7). Black arrowheads show the presence of *LacZ* signals at the frontal cortex corresponding to *CDH10* expression in human fetal brain^4^. The boxed area in P7 sagittal section is used for immuno-staining in (**c**). Scale bar, 1 mm for whole brain and sagittal section. (**c**) The βGal expressing forebrain neurons in Human BAC4 Tg also express AUTS2, a well-known marker for the frontal identities. Scale bar, 100 μm.

To probe the enhancer regulating the frontal *CDH10* expression, we selected additional Human BAC4 covering the 5′ upstream region of *CDH10* and generated transgenic mice for enhancer trapping (Fig. 6a). Consequently, we found that Human BAC4 Tg captured the human specific *CDH10* expression pattern enriched in the forebrain^4^ at the early developmental stages (from P0 to P7, Fig. 6b). Furthermore, we confirmed that the βGal expressing forebrain neurons in Human BAC4 Tg also expressed AUTS2, a well-known marker for frontal identities (Fig. 6c). Thus, contrary to our expectation that 5p14.1 ASD-associated SNPs might reside within a long distant enhancer for the *CDH10* expression at early developmental stages, we concluded that a forebrain enhancer for *CDH10* mainly resides in the vicinity of the gene.

## Discussion

All of the common variants (SNPs) associated with ASD in GWAS studies thus far are located in intergenic regions^4^,^20^ or intronic sequences^21^, outside the protein coding regions. Regarding one of these associations, Wang *et al.* speculated that the 5p.14.1 signals were capturing the functional variants that regulate the neighboring gene expressions^4^. It is noteworthy that the data list reported by Maurano *et al.* contained two 5p14.1 ASD-associated SNPs (rs4307059 and rs10038113) resided in DNaseI hypersensitive sites, the markers for altered chromatin structures by transcription factors’ bindings, supporting the hypothesis that these risk SNPs might be regulatory variants^22^. In our present study, we performed a Human BAC scan approach to examine the 5p14.1 gene desert for regulatory activities and demonstrated that rs4307059 and five other SNPs fall within *in vivo* brain enhancers which promote non-coding RNA expressions in the cortical areas, striatum, and cerebellum (Fig. 5d, Fig. S1). We also showed that the functional non-coding RNA, *MSNP1AS*, was indeed transcribed exogenously in our Human BAC2 Tg mice harboring the ASD-associated locus (Fig. 4b–d). Due to the lack of postmortem brain samples during developmental stages, it is extremely difficult to examine the *MSNP1AS* expression directly in Human fetus/neonate brains, highlighting the value of our results. Although Kerin *et al.* tried quantifying *MSNP1AS* by using a purchased total RNA from a human fetal cerebral cortex, the antisense RNA transcript was not detected^7^. By applying the BAC transgene copy number assay using a LacZ probe to the genomic DNA^23^, we confirmed that our Tg mice carried more than ten copies of full-length Human BAC2 corresponding to the *MSNP1* locus, much larger copies than the endogenous state. This would be the right reason why we were likely to be able to detect the antisense RNA transcripts in the developmental stages.

Encoding a member of the ERM (ezrin/radixin/moesin) family of proteins, moesin is known to link the cellular membrane proteins to the actin cytoskelton and participate in variety of cell signaling pathways. Although neither brain development nor behaviour has been examined in moesin knockout mice, accumulating evidence has already indicated that moesin functions both presynaptically to regulate actin-dependent growth cone motility^24^,^25^ and postsynaptically to induce dendritic spine formation^26^,^27^. It has been shown that *MSNP1AS* binds *MSN* mRNA transcribed from *MSN* gene on chromosome X, and its over-expression in cultured human cells causes decreases in MSN protein^7^,^17^ (Fig. 5d). As the very low levels of moesin protein were observed in the cerebral cortex of fetuses with Down Syndrome^28^, moesin dysregulation at critical developmental stages might also lead to abnormal brain development in individuals with ASD.

However, as depicted in Fig. 5d, Kerin *et al.* reported that MSN protein is unchanged in postmortem brain of individuals with ASD^7^, just like the case with our Tg mouse brain. As the machinery, they showed (1) *MSNP1AS* is increased, (2) *MSN* mRNA is also increased (slightly increased in our Tg mouse brains; Fig. 5a), (3) MSN protein is unchanged, (4) MSN protein levels were negatively correlated with the difference between *MSN* and *MSNP1AS* expression levels. From these results, they made a point that the antisense RNA could contribute to the MSN protein regulation. They also demonstrated that, in the over-expression experiment, the reduction effect was time-dependent and the reduction rate became smaller after 72 hours than 24 hours. This decreasing trend was also sometimes conflicting among experiments conducted in different cell lines. In the light of these facts, they explained the unchanged MSN protein levels in adult postmortem brains. Taken together, we interpret the absence of Msn protein reduction in Human BAC2 Tg as the similar situation to human postmortem brains. Compared to the homogeneous population in the cultured cells, heterogeneous cell assembly in the human postmortem/BAC-Tg brain might additionally minimize the decreasing trend, if any (Fig. 5d).

Additionally, in Human BAC2 Tg mice, endogenous *Msn* mRNA transcribed from *Msn* gene on chromosome X has 68.6% similarity at the maximum to *MSNP1AS* derived from the Human BAC2 transgene (see Supplementary Fig. S2), which might not be enough to reduce the *Msn* mRNA and protein levels (Fig. 5a–c). Although the transgene copy number was more than two in the Tg mice as was mentioned above, the exogenously expressed antisense RNA amount might be still lower than that of endogenous *Msn* mRNA. Indeed, expression levels of *MSNP1AS* quantified in Fig. 4b–d tended to be lower than those of *Msn* mRNA determined in Fig. 5a, whereas the antisense RNA levels were relatively similar to the *MSN* mRNA levels in human postmortem cerebral cortex^7^. These two reasons, the lower matching possibility and the lower expression level in the heterogeneous tissue, might also explain why Msn protein amount was not affected in Human BAC2 Tg (Fig. 5d).

For the future steps, it should be needed to clarify whether or not this Tg line is behaviorally normal by utilizing the general criteria including social behavior tests. In addition to the current standard behavioural phenotyping assays for mouse models of autism^29^,^30^, more sensitive measures and techniques that have been developed recently to catch very subtle differences in ASD cases^31^ might be applied to our Tg mice. Genome editing methodology could further allow to replace mouse *MSN* region with human sequences, strengthening and/or humanizing the Tg phenotype.

Noticeably, the *MSNP1AS* was reported to be significantly increased in individuals with the ASD-risk genotypes. For example, *MSNP1AS* expression was 22.0-fold higher in individuals with the ASD-associated rs4307059 T/T genotype compared to the non-risk C/C genotype^7^. As we confirmed that all the six SNPs associated with ASD in Human BAC2 were risk versions, the enhancer activities we detected were for the risk genotypes. Future work may investigate whether the risk genotypes have stronger enhancer activities relative to the non-risk genotypes or not.

It is reported that long non-coding RNAs (lncRNAs) often show precise temporal and spatial patterns of expression in human brains^32^,^33^,^34^,^35^, and particularly, the differential expression of lncRNAs in postmortem ASD brains was reported^36^. Although the sample size was relatively small, the data showed that some lncRNAs were unique to the prefrontal cortex while other lncRNAs were abundant in the cerebellum^36^. Given the roles of lncRNAs in brain development and function, their dysregulation can stand as contributing factors to neurodevelopmental disorders^17^,^37^,^38^. The spatio-temporal enhancer activities for a lncRNA *MSNP1AS* hence indicate the possible connection of this transcript to the disease susceptibility.

As we showed in Fig. 1b, *MSNP1AS* transcript is primate specific. Notably, the majority of lncRNAs are not conserved in comparison to the prevalence of conserved protein-coding genes^39^,^40^. Considering this with the hypothesis that human specific lncRNA could be linked to human complexity and disorders^17^,^38^,^41^, the ASD-associated interval might simultaneously include *MSNP1AS* enhancers delineating the human traits.

Collectively, the 5p14.1 ASD-associated interval possesses a dual aspect, the presence of a functional non-coding RNA and the *cis*-regulatory elements that control its spatio-tamporal expression. ASD-associated SNPs in this locus might therefore assert their influence on disease susceptibility by affecting brain enhancer activities for *MSNP1AS*.

Our study represents one of the efficient applications of *in vivo* Human BAC Tg strategy as a “post-GWAS tool” for non-coding variants to scan *cis*-regulatory activities implicated in complex diseases. Since common variants have more subtle effects on pathogenesis than rare variants, it has been extremely challenging to model the effect in animals. In this context, our results provide valuable information as a part of worldwide efforts to interrogate the complex genetic architecture of ASD, and help elucidate the pervasive involvement of regulatory variations in the common disease.

## Method

### Human and Mouse BAC clones used in this study

Human BAC1 (RP11-140M9), Human BAC2 (RP11-261G10), Human BAC3 (RP11-99D18), Human BAC4 (RP11-2G19), and Mouse BAC2 (RP23-225M16) were purchased from BACPAC Resources (CHORI). By sequencing, all the six SNPs associated with ASD risk in Human BAC2 were confirmed to be major version (risk version). For instance, rs4307059 was common T version, and the five others in linkage disequilibrium (LD) block were also common version. UCSC Genome browser was used to annotate the BAC end positions and SNP locations in Fig. 1.

### Bioinformatic analyses for evolutional conservations

A web-based tool “ECR browser” (ecrbrowser.decode.org) was employed to compare multiple vertebrate genome sequences corresponding to ASD GWAS SNP region. A vertical axis cut-off of 50% to 100% identity was utilized to visualize the significant conservation (Fig. 1b).

### Transposon-mediated BAC modification

All the BACs were modified by *in vitro* random transposition of Tn1000 βglobin-LacZ^9^ and the transposon insertion sites were determined by sequencing as described previously^42^.

### Generation of BAC transgenic mice

All animal care and experiments were performed in accordance with the Guidelines for Animal Experiments approved by the Animal Care and Use Committee of the National Institute of Neuroscience, Japan (approval number: 27-21). BAC DNA was purified by using Nucleobond BAC 100 (Macherey-Nagel), linearized with PI-*Sce*I (NEB), and microinjected into pronuclei of B6C3F1 (SLC, Japan) fertilized eggs at the concentration of 1.2 ng/μl as detailed previously^42^. The presence of the BAC vector end sequences was always confirmed by PCR using the same primers as was previously^43^ to eliminate the possible fortuitous deletions. Multiple independent transgenic lines were generated for each BAC clone, and the representative LacZ expression patterns confirmed by multiple lines were shown in the figures. Human BAC2 Tg line (line No. 3) had been maintained in B6C3F1 background for developmental LacZ expression pattern analyses, RNA quantification assays, and Western blot analyses.

### Detection of β-galactosidase activities

E12.5 embryos and E17.5 to P56 brains were harvested from BAC transgenic mice. Detection of β-balactosidase (βGal) activities was performed in whole-mount samples or in sliced brains as previously described^42^.

### Immunohistochemistry for the brain sections

Immunostaining was performed according to the standard methods. Primary antibodies and dilutions used were chicken anti-beta galactosidase (βGal, 1:3000, abcam ab9361), rabbit anti-Cux1 (1:3000, Santa Cruz Biotechnology sc-13024), goat anti-Bhlhb5 (1:3000, Santa Cruz Biotechnology sc-6045), rabbit anti-RORβ (1:6000, Diagenode pAb-001–100), mouse anti-NeuN (1:500, Millipore MAB377), mouse anti-GAD67 (1:500, Millipore MAB5406), rabbit anti-Zic2 (1:100, provided by Dr. Jun Aruga^44^), and rabbit anti-AUTS2 (1:300, ATLAS antibodies HPA000390). Appropriate secondary antibodies were from the Molecular Probes Alexa series and used at dilutions of 1:600.

### Quantitative reverse-transcription PCR

Total RNA was extracted from wild-type and BAC transgenic mouse brain tissues by using Trizol Regent (Invitrogen) and High Pure RNA Tissue Kit (Roche), as described in the manufacturer’s protocols. cDNA was generated by the use of ReverTra Ace qPCR RT Master Mix with gDNA Remover (TOYOBO) according to the manufacturer’s protocol. qPCR assays were performed with TaqMan Gene Expression Assays (Applied Biosystems) running on a ABI 7900HT system and the data was analyzed with SDS2.1 software. The probe for *MSNP1AS* had already been designed by Campbell’s Lab^7^ to target the most divergent sequence between *MSNP1AS* and the mature *MSN* transcript, and was custom-synthesized by Applied Biosystems. TaqMan Gene Expression Assay for *Msn* (assay ID Mm00447889_m1), *LacZ* (assay ID Mr00529369_cn) and *Gapdh* (assay ID Mm99999915_g1) were also purchased from Applied Biosystems. All results presented represent normalization to *Gapdh* (glyceraldehyde-3-phosphate dehydrogenase) expression.

For each time point (E17.5-P7) and each brain structure (somatosensory cortex, striatum, cerebellum), 4 independent mouse brain tissues were used to extract total RNA. All qPCR reactions were performed in triplicate. Pearson correlation coefficient (*r*) and *P* value between *LacZ* mRNA expression and *MSNP1AS* RNA expression were calculated by using Microsoft Excel 2011 statistical tools.

### Western blotting

Total proteins were extracted from wild-type and Human BAC2 Tg mouse brains and separated by 10% polyacrylamide gel electrophoresis. Proteins were transferred to PVDF membrane (Millipore IPVH00010) with AE-6687 HorizeBLOT system (ATTO). Primary antibodies and dilutions used were rabbit anti-Moesin (Msn, 1:1000, Cell Signaling 3146) and rabbit anti-GAPDH (14C10, 1:2000, Cell Signaling 2118). Secondary antibody was anti-rabbit IgG, horseradish peroxidase-linked (GE Healthcare NA9340V) diluted 1:5000. Signal bands were detected by using ECL Prime Western Blotting Detection Regent (GE Healthcare RPN2232) and images were captured on LAS-4000 Luminescent Image Analyzer (Fujifilm). Total density of each Msn and Gapdh protein band was determined with ImageJ (version: 2.0.0) software. For each sample, the ratio of Msn to Gapdh total density was calculated. Msn-to-Gapdh ratios were analyzed by Student’s *t* test for comparison of wild-type to Human BAC2 Tg brain samples (n = 3 for each brain regions).

## Additional Information

**How to cite this article**: Inoue, Y. U. and Inoue, T. Brain enhancer activities at the gene-poor 5p14.1 autism-associated locus. *Sci. Rep.* **6**, 31227; doi: 10.1038/srep31227 (2016).

## Supplementary Material

Supplementary Information
