# Supplementary material for: Brain enhancer activities at the gene-poor 5p14.1 autism-associated locus
Source: Sci Rep. 2016 Aug 9;6:31227. doi: 10.1038/srep31227 (PMC4977510; doi:10.1038/srep31227)
Supplement: Supplementary Information [file srep31227-s1.pdf]

Supplementary information

Brain enhancer activities at the gene-poor 5p14.1 Autism-associated locus

Yukiko U. Inoue* and Takayoshi Inoue

Department of Biochemistry and Cellular Biology, National Institute of Neuroscience, National Center of Neurology and Psychiatry, Ogawahigashi, 4-1-1, Kodaira, Tokyo 187-8502, Japan

*Author for correspondence
E-mail address; yinn3@ncnp.go.jp

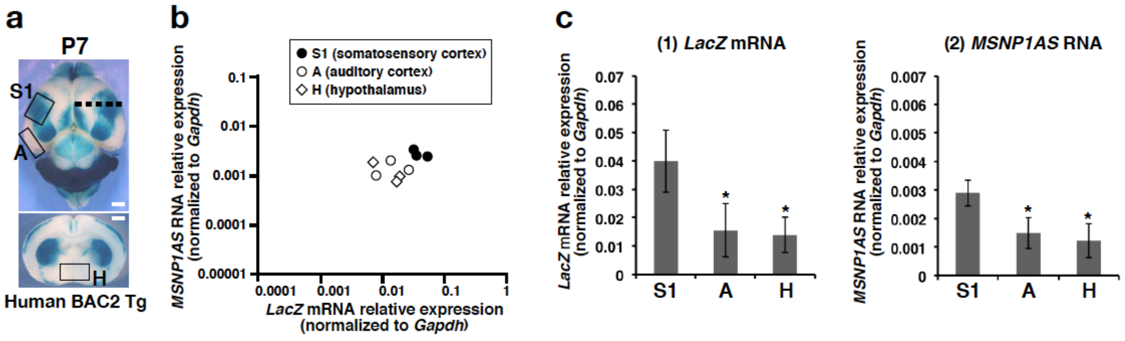

Supplementary Figure S1

Brain region-specific enhancer activities for *MSNP1AS* in Human BAC2 Tg mouse

In addition to the *LacZ* blue-stained brain region (S1; somatosensory cortex), we sampled two brain regions with little *LacZ* blue-staining at P7 (A; auditory cortex, H; hypothalamus) as the reference and quantified the *LacZ* mRNA/*MSNP1AS* expression levels.

(a) The upper panel shows the dorsal view of the whole-mount *LacZ* stained brain from Human BAC2 Tg at P7. The lower panel shows the coronal section at the black dotted line in the upper panel. The brain tissues corresponding to the boxed regions are sampled for total RNA extraction (n=3 for each region). S1; somatosensory cortex, A; auditory cortex, H; hypothalamus. Scale bars, 1 mm for whole brain and coronal section.

(b) *LacZ* mRNA/*MSNP1AS* expression levels quantified by RT-PCR. The transcript levels in the blue-stained somatosensory cortex (S1) were higher than those in the non-stained auditory cortex (A) and hypothalamus (H), i.e. the transcription was enhanced in blue-stained regions.

(c) (1) Quantitative summary of *LacZ* mRNA expression levels in (b). The transcript level in the blue-stained S1 was higher than those in the non-stained A and H. $*P < 0.05$ by Student's *t* tests. (2) Quantitative summary of *MSNP1AS* expression levels in (b). The transcript level in the blue-stained S1 was higher than those in the non-stained A and H. $*P < 0.05$ by Student's *t* tests.

Supplementary Figure S2

Mouse *Msn* mRNA has 68.6% similarity to *MSNP1AS* at the maximum.

Mouse *Msn* mRNA sequences (NM_010833.2) in the upper row are aligned with *MSNP1AS* sequences (NT_006576.16) in the lower row. The maximum matching possibility is figured out as 2,706/3,940 nucleotides (= 68.6%).

Supplementary Figure S2

Mouse *Msn* mRNA has 68.6% similarity to *MSNP1AS* at the maximum.

```
Alignment of Sequence_1: [Mouse Msn mRNA (NM_010833.2)]
        with Sequence_2: [MSNP1 (NT_006576.16) antisense]

The maximum matching possibility: 2,706/3,940 nucleotides (= 68.6 %)

Seq_1  1      ---------TCTTTCCTGGGTGGGTTTGTAAAGTCGTGGCCCGTTAGCAGGAAGCGGAG  51
Seq_2  1      agaggcggctcttgcctgggtggggtttgtgaagtcgtggcccgttagcaggaagcctaa  60

Seq_1  52     CAGTCGCCCA-ACGCTAGAGTGGGACCCAATCTGAGCACCCGGTTCTCCGGAGTCCTTGC  110
Seq_2  61     cagtcgccccgacgctagtgagggacccaatctgagtccccggccagccgaatccca-gc  119

Seq_1  111    CGAAGTGTACTGCGCGCTCAGAGCAGCCCGACAGTTTCCAGCCAAACTTCACCAACTCCT  170
Seq_2  120    cgt-gtgtactgcatgctcagcactgcccgacagtc-ctagctaaacttcgctaactccg  177

Seq_1  171    CTGCCCTTGCCGCCACCATGCCGAAGACGATCAGTGTGCGTGTCACCACCATGGATGCAG  230
Seq_2  178    ctgcctttgccgccaccatgcccaaaacgatcagtgtgcgtgtgaccaccatggacgcag  237

Seq_1  231    AGCTGGAGTTTGCCATTCAGCCCAACACCACTGGCAAGCAGCTGTTTGACCAGGTGGTGA  290
Seq_2  238    agctggagtttaccatccagcccaacaccaccgggaagcagctatttgaccaggtggtga  297

Seq_1  291    AAACTATTGGTTTGAGGGAAGTTTGGTTCTTTGGTCTGCAGTACCAGGACACAAAAGCTT  350
Seq_2  298    aaactattggcttgagggaagtttgtttctttggtctgcagtaccaggacaccaaaggtt  357

Seq_1  351    TCTCTACTTGGCTGAAACTCAAT-AAGAAGGTGACTGCACAGGATGTGCGGAAGGAAAGT  409
Seq_2  358    tctccacctggctgaaactcaattaagaaggtgactgcccaggaagtgcggaaggaaagc  417

Seq_1  410    CCATTGCTCTTCAAGTTCCGGGCCAAGTTCTACCCAGAGGATGTATCTGA---A-GAACT  465
Seq_2  418    cccctgctctttaagttcagtgccaagttctaccctgaggatgtgtccgaccgaggaatt  477

Seq_1  466    GATCCAGGATATCACCCAGCGCCTGTTCTTTCTGCAAGTGAAGGAGGGCATTCTCAATGA  525
Seq_2  478    gattcaggacaccactcagcgtctgttccttctgcaagtgaaagagggcattctcagtga  537

Seq_1  526    CGACATTTACTGTCCACCTGAAACTGCGGTCCTGTTGGCTTCTTATGCCGTCCAGTCTAA  585
Seq_2  538    tgatatttactgccggcctgagactgctgtgctgctggcctcccatgctgtccagtctaa  597

Seq_1  586    GTATGGTG-ACTTCAATAAGGAAGTGCACAAGTCTGGCTACCTGGCTGGAGATAAGTTGC  644
Seq_2  598    gtatgg-gaacttcagtaaggaggtgcataagtctggctacctggccggagacgagttgc  656

Seq_1  645    TTCCCCAAAGAGTCTTGGAGCAGCACAAACTCAACAAGGACCAGTGGAAGAGAGGATCC  704
Seq_2  657    tcccacagagagtcctggaacagcacaaactcagcaaggaccagtgggaggagcggatcc  716

Seq_1  705    AGGTGTGGCATGAGGAGCACCGGGGCATGCTCAGGGAGGATGCTGTCCTGGAATATCTCA  764
Seq_2  717    aggtgtggcatgaggaacaccgtggcatgctcagggaggatgctgtcctggagtatctga  776

Seq_1  765    AGATTGCTCAAGACCTGGAAATGTATGGTGTGAACTATTTCAGCATCAAGAACAAGAAAG  824
Seq_2  777    agattgctcaagatctggagatgtatggtgtaaactacttcagcatcaagaaaaagaaag  836
```

```
Seq_1  825   GCTCAGAGCTATGGCTGGGCGTGGATGCCTTGGGTCTCAACATCTATGAGCAGAATGACA  884
             |||||||||| |||||||| ||||||||  ||||||||||||||||||||||| ||||||
Seq_2  837   gctcagagctgtggctgggggtggatgcactgggtctcaacatctatgagcagcatgaca  896

Seq_1  885   GACTGACTCCTAAGATTGGCTTCCCGTGGAGTGAAATCAGGA--ATATCTCTTTCAATGA  942
             |||| ||||| ||||| ||||| || ||||||||||||||||  || | |||||||||||
Seq_2  897   gactaactcccaagataggctttccttggagtgaaatcaggagcat-t-tctttcaatga  954

Seq_1  943   TAAGAAATTTGTCATCAAGCCCATTGACAAAAAGGCCCCGGACTTTGTGTTCTATGCTCC  1002
             |||||| |||||||||||||||||||||||||  |||||||||||||| |||||||||||
Seq_2  955   taagaactttgtcatcaagcccattgacaaaagagccccggactttgtcttctatgctcc  1014

Seq_1  1003  CCGGCTTCGGATTAACAAGCGGATCTTGGCCCTGTGCATGGGAAATCATGAGCTGTACAT  1062
             | |||| | |||||||||||||||||||||| |||||||||| || | ||| || |||||
Seq_2  1015  ctggctgcagattaacaagcggatcttggccttgtgcatggggaaccgtgaactatacat  1074

Seq_1  1063  GCGTCGGCGCAAGCCTGACACCATTGAGGTGCAGCAGATGAAGGCCCAGGCTCGGGAAGA  1122
             ||| || |||||||||||||||||||||||||||||||||||||| | ||| ||||| ||
Seq_2  1075  gcgccgtcgcaagcctgacaccattgaggtgcagcagatgaaggcacgggcccgggagga  1134

Seq_1  1123  GAAGCACCAGAAGCAGATGGAGCGTGCTCTCCTGGAAAATGAGAAGAAGAAGCGTGAGCT  1182
             |||||||||||||||||||||||||||| | ||||||||||||||||||   |||||  |
Seq_2  1135  gaagcaccagaagcagatggagcgtgctatgctggaaaatgagaagaag---cgtgaaat  1191

Seq_1  1183  GGCTGAGAAAG-AGAAGGAGAAGATTGAGCGGGAGAAGGAAGAGCTGATGGAGAAGCTGAA  1242
             ||| || |||| |||| |||||| |||| ||||||||||| |  ||||||| || ||||||
Seq_2  1192  ggcaga-aaagcagaaagagaagattgaacgggagaagga-g--ctgatggggaggctgaa  1248

Seq_1  1243  GCAGATTGAGGAGCAGACTAAGAAGGCTCAGCAAGAGCTGGAAGAGCAGACCCGCAGGGC  1302
             |||||| ||||| ||||||||||||||||||||||| |||||||| |||||||| |||||
Seq_2  1249  gcagatcgaggaacagactaagaaggctcagcaagaactggaagaacagacccgtagggc  1308

Seq_1  1303  CCTAGAACTTGAGCAGGAACGGAAGCGTGCCCAGAGTGAGGCCGAAAAGCTAGCCAAGGA  1362
              || ||||||| | |||||||||||| ||||||||| ||||| |||||||| ||||||||
Seq_2  1309  tctggaacttgggaaggaacggaagcatgcccagagcgaggctgaaaagctggccaagga  1368

Seq_1  1363  GCGTCAAGAAGCTGAAGAAGCCAAAGAGGCCCTGCTGCAGGCTTCTCGGGACCAGAAGAA  1422
             || ||||||||||||||| ||||| || | | |||||||||| || ||||||||||| ||
Seq_2  1369  gcttcaagaagctgaagaggccaaggaagtcttgctgcaggcctcccgggaccagaaaaa  1428

Seq_1  1423  GACCCAGGAACAGCTGGCTTCAGAAATGGCAGAGCTGACGGCACGGATCTCCCAGTTGGA  1482
             ||| |||||| ||||||| |  ||||||||||||||||| || |  ||||| ||| ||||
Seq_2  1429  gactcaggaatagctggccttggaaatggcagagctgacagctcaaatctctcagctgga  1488

Seq_1  1483  AATGGCTCGAAAGAAGAAGGAAAGTGAGGCTGTGGAATGGCAGCAAAAGGCCCAGATGGT  1542
             | |||| ||| ||||||| || ||||||||| |||| |||||||| ||||||||||||||
Seq_2  1489  actggcccgacagaagaaagagagtgaggctatggagtggcagcagaaggcccagatggt  1548

Seq_1  1543  ACAGGAAGACTTGGAGAAGACTCGTGCTGAGCTGAAGACTGCCATGAGTACACCTCATGT  1602
             ||||||||||||||||||||| |||||||||||||||||||||||||||||| ||||||||
Seq_2  1549  acaggaagacttggagaagacccgtgctgagctgaagactgccatgagtacatctcatgt  1608

Seq_1  1603  GGCAGAGCCTGCTGAGAATGAACATGATGAGCAGGATGAGAATGGAGCAGAGGCCAGTGC  1662
             ||||||||||||||||||||| || |||||||||||||||||||| |||||||| |||||
Seq_2  1609  ggcagagcctgctgagaatgagcaggatgagcaggatgagaatggggcagaggctagtgc  1668

Seq_1  1663  CGAGCTGCGGGCTGATGCTATGGCCAAGGACCGCAGTGAGGAGGAACGTACCACTGAGGC  1722
              || || ||||||||||||||||||||||||||||||||||||||| |||||||||||||
Seq_2  1669  tgacctacgggctgatgctatggccaaggaccgcagtgaggaggaatgtaccactgaggc  1728

Seq_1  1723  AGAGAAGAATGAGCGTGTGCAGAAGCATCTGAAGGCCCTTACTTCAGAGCTGGCCAATGC  1782
             ||||||||||||||||||||||||||| ||||||||||| ||||| |||||||||| |||
Seq_2  1729  agagaagaatgagcgtgtgcagaagcacctgaaggccctcacttcggagctggccagtgc  1788
```

```
Seq_1  1783  CCGAGATGAGTCCAAGAAGACTGCCAATGACATGATCCATGCTGAGAACATGCGACTGGG  1842
             |||||||||||||||||||||||||||||||||||||||||||||||||||| |||||||
Seq_2  1789  ccgagatgagtccaagaagactgccaatgacatgatccatgctgagaacatgtgactggg  1848

Seq_1  1843  ACGAGACAAATACAAGACCCTGCGCCAGATCCGGCAGGGCAACACCAAACAACGCATTG-  1901
              ||||| ||||||||||||||| ||||||| || ||||||| |   || ||| |||
Seq_2  1849  ccgagataaatacaagaccctgtgccagat-cgtcagggcagc---aa-caa-gcagcac  1902

Seq_1  1902  AT-GA-G--TTTGAGTCCATGTAGTGGGCGTCCAGCCTTCAGGGACCCCTCCTCCTTCTTCCT  1960
             || || |  |||||||| | ||| |||||  |||||||  ||||||| ||||||| | || |
Seq_2  1903  attgacgaatttgagtctaagtaatgggcacccagcctctagggaccgctcctccctttttcc  1965

Seq_1  1961  TGTCCCCACACTCCCATAGTT---TTGCCTAACTAACACTGTGCTGGAGCCACTAACTAGAAA  2020
             |   |||||||||  | |  |   |  ||||||| | |||||||||||| |||||||||||
Seq_2  1966  ttgtcccacactcttacacctaactcacctaactcatactgtgctggagtcactaactagagc  2028

Seq_1  2021  AGCCTTGGAGCCATGCCAAACGTTCAGTATAGCCATGGGACCAAACTTAATCTCCCCACTCAT  2083
             |||| ||||| |||||||| | || ||| ||||||||||||||  | ||  | |    | |
Seq_2  2029  agccctggagtcatgccaagcatttagtgtagccatgggaccagccctagacccttagccccc  2091

Seq_1  2084  ACCC---TC---GGGCAAACAAATGGCCCACTGTGGTGCCAATGGAATCTCCTTTTCCTTCTT  2140
             ||||   ||   |||||||  |||||| |||| |||||||||||||| |||||||  | |||
Seq_2  2092  acccacttccctgggcaaatgaatggctcactatggtgccaatggaacctcctttctcatctc  2154

Seq_1  2141  TGTCACACTCATTCAACCTAGCTCTCTAGAATAGAGCATTTCTCCCCGCCCCAGCTCAGA  2200
             |||  |   |||||||||||  |  ||||||||      |||  |     ||||| ||
Seq_2  2155  tgt-tc---catttaatctgtgttgctagaata------ttccacttct-ccagccca--  2201

Seq_1  2201  GACACGCATTCCTTTTGGTTTGACAAGCACCCCCTCCCCATATACACTTACTGTTGTCCT  2260
             ||    | |||| |||| |||  |||  ||||  |       ||||||||||||||||||
Seq_2  2202  gaggtactttccatttgattttgcaaataccc--t-------tacacttactgttgtcct  2252

Seq_1  2261  CTGGGACTCATGTGTGAAGTAGGTTAACAGCTAGCTCCCATCCCTTGCCAGTCCTGTG--  2318
              ||||| ||| ||||| ||||||||   ||||||||||| |||| ||||   ||  ||
Seq_2  2253  atgggagtcaagtgtggagtaggttggaagctagctcccctcccctcccctaccactgcc  2312

Seq_1        ------------------------------------------------------------

Seq_2  2313  ttcttcttcagggtcctgagatttacagggttggagtgttttgcggtttagggaatgaga  2372

Seq_1  2310  ------------------------------------------------------------

Seq_2  2373  caggacctt-gg-atatctgctccaggctgtcaattaacctaaaatttgtcctcccagtg  2430

Seq_1  2319  ---ATTTGCAAAGATGGAATATTGTGTTGTTTAGGGACAGAAGGAGGGAATTGGCTTTAT  2375
                ||          |  || | |  ||  | | |    |  |   | ||     |   |
Seq_2  2431  cccatcccgtttatagttatttaggctttgtaatgactggggatagaaagatgttcagt  2490

Seq_1  2376  CATTTAG-TATGTCAGATGACCTAGGATTTAGCCTCTTAATTAAGTCTATCCCTTTTACA  2434
             |||||   | | |                  |      |   |     |       |
Seq_2  2491  cattttatttctacctcccagattggacctgttacaaactcagcctcaataagccttgt  2550

Seq_1  2435  GTTACTTAGGCTTTGTACCGATTGGAGGATAAAAGAGATGTTTAGCCATTCTTATTTCTT  2494
               |              |   ||           |    |   |  |  |    |
Seq_2  2551  tgttgactttagggactcaatttctccccaggtggatggggaaacggtgccttcaaga  2610

Seq_1  2495  CTACCTCCCATATTGGACCTATTACAAATCTAGTCCCAGTGGGCCTTGTCTCTGTGTTTA  2554
                    ||| ||       |   | | |       |  |                   |
Seq_2  2611  ggcttcaccaaatgtactagaaggccattggccattctattctggcaaggctgagtagaa  2670

Seq_1  2555  GGGATTTAGTTTCTAATCAAGATGGGATGAAGAGATAAAGGAAATAGTGCATTCAAGAGA  2614
             |    |       |  |  | | | |   | |         |   ||  |  |    |||
Seq_2  2671  gatcctaccccaattctttacaggagtataggcctgtctaaagtgagctccatgggcaga  2730
```

```
Seq_1  2615  TTCACTAACAACCTAGAAAGGCCCCTGCCCCAGCACATCTTTGGAGCCTTTCTTCTCTTG  2674
Seq_2  2731  gctaccccttattattccggacctgcagtcacttcgtgggatctgcccctccctgcttca  2790

Seq_1  2675  AGTGAGGTCTGTGGTCAGGGCTACCCCTGCCCATGATAGTCCAGGTTCTTGGTTCCTTTT  2734
Seq_2  2791  gtacccaaatcctttccagctataacagtagggatgagtacccaaaagctcagccagccc  2850

Seq_1  2735  CTAAATCTGTCCCTACCCTCTCCAATCCTCAGATCTCTTCCAGCTAGGACAGAGAGGATG  2794
Seq_2  2851  catcaggattcttgtgaaaagagaggatatgttcacacccagcttcagtattttccctgc  2910

Seq_1  2795  AGTACCCTCAAACCTAGCCAGTTGCATCTAAACTTGTTGCCACAAAGGAAGAAACTTTTT  2854
Seq_2  2911  caggagttttaggtctcttcctctctcagagctacttgggctatagctcctgctccacag  2970

Seq_1  2855  TGTACCTGGCTTTAGTATTTTTCCTGCCAGGACTTCTAAACTACTTCCCCCAGAGCTAAT  2914
Seq_2  2971  ccatcccggccttggcatctagagcttgatgcctgtaggctcaactagggagtgagtgca  3030

Seq_1  2915  TTGACCACTGTTCCTGTTTCACAGATACAAAATATGATGAGTAAGAGTAGCCCTTGTCTGTC  2974
Seq_2  3031  agaagctgagtatggtgagagaagcctgtgccctgatcccagtttactcaaccctgtcacat  3092

Seq_1  2975  AGTCACCCAGCAATCCTCCCTCATCTTACCCAGTGCAAAGTGGTGAGTAGGCCCTGC-----T  3034
Seq_2  3093  gaccaaaatccccttctcatcactc---ccc--tccaaagtggtgactaggccctgcctctgt  3150

Seq_1  3035  TCTGACAAATCTCTAACTCAGGCCTCATCACTCATTGTGCTGTCCCTTGGAGATACAGA--A-  3094
Seq_2  3151  t-tgacaaacttctaacccaggtcttgacaccagctgttctgtcccttggagctgtaaaccag  3212

Seq_1  3095  AG-CTGCTGGGTATTCTGGCCTT-GTCCCTTC-ACACCCCCACACCCTTTTCTCCACTTTGGA  3154
Seq_2  3213  agagctgcgggtaattctagcctagtcccttccacacccccacgcc--ttgct---ttcaacc  3270

Seq_1  3155  GCAGCTGCTCCCCCTTTTTTGTCACATGTGTGCTCTTTTAATTTTTAC--CAGTATTATA  3212
Seq_2  3271  caggagcctccacctccttctctgtctcatgtgtactcttcttctttctacagtgttatg  3330

Seq_1  3213  TATTCTAGTGATATCTAACAATATTGGTTTCTACCTTTTTTGCTAATGCACTATTAG-AGAT  3273
Seq_2  3331  tattctactgatacctaa--atattgatttctgtcttccttgctaatgcaccattagaagat  3390

Seq_1  3274  ATTAGTCTTGGGAAAGGATCATTTTAGCTTCATTATTTTTTACTACTGTCACCCCACATCT  3333
Seq_2  3391  attagtcttggg---g---caggatcattttggcctcattcctttaccact-cccacacct  3444

Seq_1  3334  GAGAAGAATATACTATATTA-AAAATGATATTTTGCCAAAAACATTATG-TAAGAAGATTT  3393
Seq_2  3445  ggaaagcatatactatattacaaaatgacattttgccaaaaatattaatataagaagcttt  3505

Seq_1  3394  CAGTATTAGCGACTT-ACCTGTCACTATAGGTCATACAAT--A--CATTTAAAATGTACT  3448
Seq_2  3506  ca-tattagtgatgtcatctgtcactataggtcatacaatccattc-ttaaagtacttgt  3563

Seq_1  3449  TAGTATTTGGTTTTATTGTTCCTGTTTGCTTTTTCCCCAGGGTTCAGTCCCCAAAAGGCT  3508
Seq_2  3564  t-g--tttgttttattattcctgtttgtcttctcctcagggttcagccctcaaggggcc  3620

Seq_1  3509  GTCTTGTCCCTCTATGCAGTATCCTTAGTCCAGAGCATCCCTCAGCCCATGCCCCTTTTACT  3568
Seq_2  3621  accctgtcccaccataaagagcctctagcttagaacctccctcaattccccgtccaccaccc  3682
```

```
Seq_1  3569  T--AATCT-TGGGGAATCTT-AT-A--------TGCATTGCTGTGAATTATATTG--AC--  3614
                | ||| ||    | ||| |  |        ||||||||||||||||||||||  ||
Seq_2  3683  cccactctgtgcctgaccttgaggagtcttctgtgcattgctgtgaattatattggcactc  3743

Seq_1  3615  AGTTGGTGAGCTGTCTTATATTGGCTAAATTCAAACCTGGAATTGTGGGGGCAATC-----  3670
             | |||||||  || | |||||||||||||||||||| ||||||||||||| |||||
Seq_2  3744  acttggtgatatgccctatattggctaaattcaaacttggaattgtgggg-caatctatta  3803

Seq_1  3671  -TAGCTGCATTAAGGGCAATAACCCACCCCCCCATAGAGGCTGGGGAGAAGGGTAGGATTT  3730
              ||||||| |||| | |  ||||  ||||    |  |||||||||||||| |||   ||||
Seq_2  3804  atagctgccttaaagtcagtaacttaccctt--agggaggctggggagaaaggttaaattt  3862

Seq_1  3731  -ATATTGAGGTTTTTTTTTTGTGTATTTTTGTTTTTTAAATAATTG-TTTTGGAGGGGTTT  3789
               |||| ||| ||||||| |||   |||| |  |||| || ||||| ||||||||||||||
Seq_2  3863  tttattcagggtttttttgtgtactttttggggtttttaaaaattgtttttggaggggttt  3923

Seq_1  3790  ATGCTCAATCTGTGTTCTATTTCAGTGCCAATAAAATTTAGGAAGACTTCA  3840
             ||||||||||  |||||
Seq_2  3924  atgctcaatccatgttc----------------------------------  3940
```

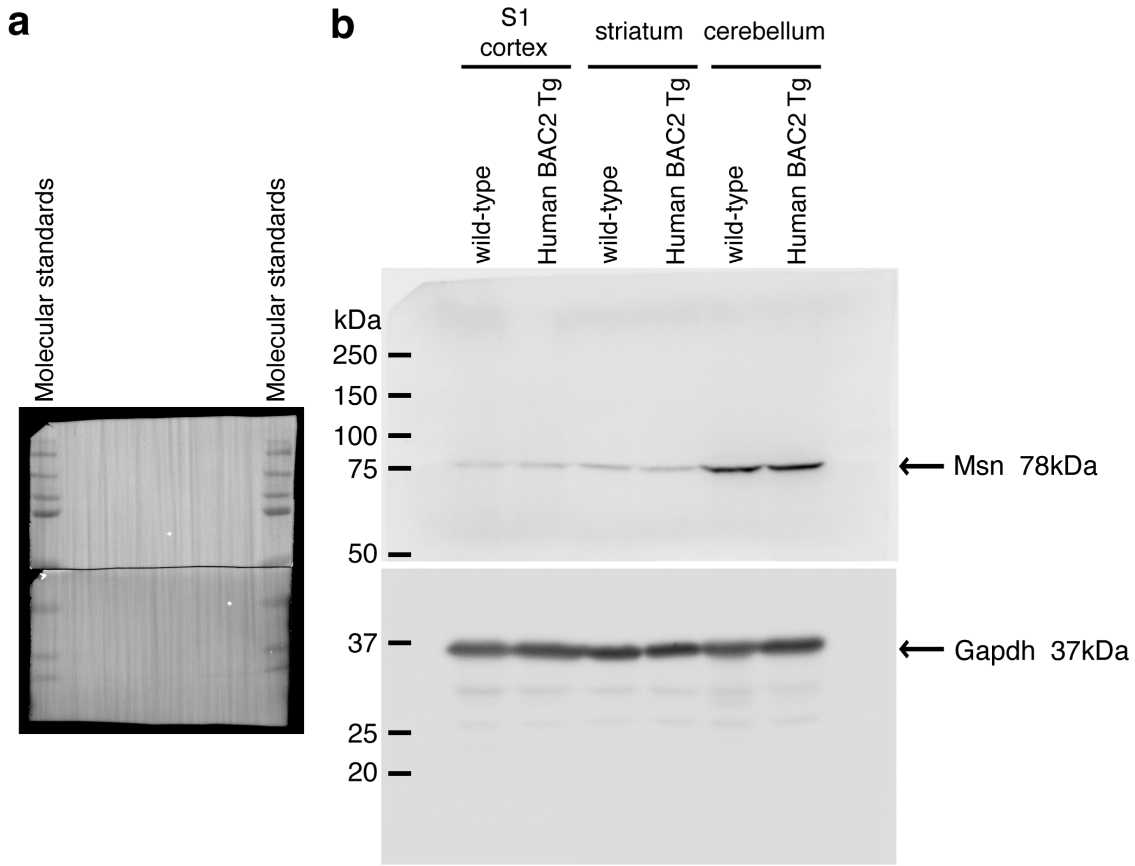

Supplementary Figure S3

Original whole image of Figure 5b (Western blotting)

(a) We cut the blotted PVDF membrane into two pieces under the 50 kilo-dalton (kDa) marker. Molecular standards; Precision Plus Protein Dual Color Standards (BIO-RAD 161-0374)

(b) The upper piece of the membrane was incubated with the Msn antibody, and the lower was reacted with the Gapdh antibody.

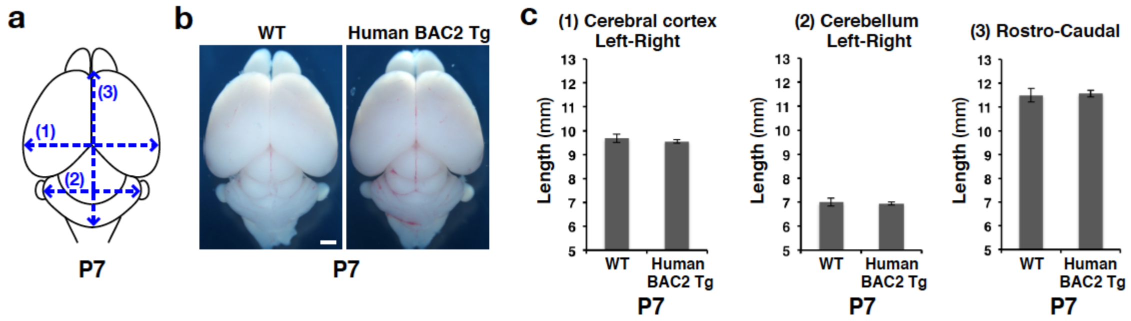

Supplementary Figure S4

Morphological measurements revealed the absence of gross abnormalities in Human BAC2 Tg mouse brains.

(a) Schematic of the P7 whole brain. Horizontal double-headed arrows indicate the maximum Left-Right length of the cerebral cortex (1) and cerebellum (2) measured by ImageJ software. Vertical double-headed arrow (3) indicates the measured maximum Rostro-Caudal length between the cerebral cortex and cerebellum.

(b) Representative whole brain images (dorsal views) for wild-type (WT) and Human BAC2 Tg mouse at P7. Scale bar, 1mm.

(c) Quantitative summary of (a) and (b). There are no significant differences between wild-type mouse and Human BAC2 Tg brains (n=4 for each genotype) by Student's *t* tests.
